# Dopamine affects short-term memory corruption over time in Parkinson’s disease

**DOI:** 10.1038/s41531-019-0088-2

**Published:** 2019-08-05

**Authors:** Sean James Fallon, Matthew Gowell, Maria Raquel Maio, Masud Husain

**Affiliations:** 1 Department of Experimental Psychology, University of Oxford, Oxford, UK; 2 National Institute for Health Research Bristol Biomedical Research Centre, University Hospitals Bristol NHS Foundation Trust and University of Bristol, Bristol, UK; 3 Nuffield Department of Clinical Neurosciences, John Radcliffe Hospital, Oxford, UK

**Keywords:** Human behaviour, Computational neuroscience, Biomarkers, Cognitive ageing

## Abstract

Cognitive deficits are a recognised component of Parkinson’s disease (PD). However, particularly within the domain of short-term memory, it is unclear whether these impairments are masked, or caused, by patients’ dopaminergic medication. The effect of medication on pure maintenance in PD patients has rarely been explored, with most assessments examining maintenance intercalated between other executive tasks. Moreover, few studies have utilised methods that can measure the quality of mental representations, which can enable the decomposition of recall errors into their underlying neurocognitive components. Here, we fill this gap by examining pure maintenance in PD patients in high and low dopaminergic states. Participants had to encode the orientation of two stimuli and reproduce these orientations after a short (2 s) or long (8 s) delay. In addition, we also examined the performance of healthy, age-matched older adults to contextualise these effects and determine whether PD represents an exacerbation of the normal ageing process. Patients showed improved recall OFF compared to ON their dopaminergic medication, but only for long-duration trials. Moreover, PD patients OFF their medication actually performed at a level superior to age-matched controls, indicative of a paradoxical enhancement of memory in the low dopaminergic state. The application of a probabilistic model of response selection suggested that PD patients made fewer misbinding errors in the low, compared with high, dopaminergic state for longer-delay trials. Thus, unexpectedly, the mechanisms that prevent memoranda from being corrupted by misbinding over time appear to be enhanced in PD patients OFF dopaminergic medication. Possible explanations for this paradoxical effect are discussed.

## Introduction

Parkinson’s disease (PD), even in the absence of dementia, is now known to be associated with a variety of cognitive control deficits, particularly in the domains of planning,^1,2^ attention,^3^ working (short-term) memory^4–6^ and learning.^7,8^ Impaired performance on these processes is commonly assumed to result from dopaminergic denervation of the basal ganglia.^9,10^ However, PD is also associated with widespread pathology involving other neurotransmitter systems: serotonergic, cholinergic and noradrenergic.^11–13^ Disruptions to the integrity of these non-dopaminergic systems may also be responsible for behavioural abnormalities in patients.^14^

Researchers have attempted to isolate the role of dopamine in causing cognitive control impairments in PD by testing patients ON and OFF their dopamine-enhancing medication.^15–18^ In the field of working memory (WM), these studies have produced conflicting results. Some report no effect of medication,^19^ whereas others have found both impaired^20–22^ or even improved^18^ performance. Researchers have attempted to account for these divergent findings by hypothesising that dopaminergic medication can have separate, possibly opposing effects, on different mnemonic subprocesses.

One prominent idea is that dopaminergic medication has different effects on maintenance versus manipulation of items in WM.^23^ This proposal has recently received support and elaboration. PD patients were found to have an enduring, dopamine-independent deficit in maintaining information over longer retention periods. In contrast, impairments in the proficiency of manipulating information (specifically updating WM contents or protecting them from distractors—ignoring) were found to be ameliorated by dopaminergic medication.^24^ Moreover, by using delayed, analogue report measures—in which the features of a memoranda (e.g., orientations) need to be reproduced—it was possible to apply a computational model to uncover the source of these errors.

In that model,^25^ errors in recall can result from changes in the precision which memoranda are stored, misbinding features belonging to different items (a form of interference) or guessing (chance). Misbinding errors occur when the different components of memoranda are erroneously combined, e.g., after encoding a red triangle and a blue square, the square is reported as being red. In contrast, guessing (or chance) errors occur when the reported memoranda bear no statistical relationship to the studied items. Application of this model to the data from patients tested ON and OFF their medication revealed that dopamine and PD affected different components in a task-dependent manner.

Withdrawing patients from their medication impaired ignoring and updating through increasing the number of guess responses. Whereas, independent of medication state, PD patients had a decrement in precision of recall for longer retention periods. Thus, these findings suggest that having PD affects the maintenance of information through increasing decay across time, whereas being on dopamine allows mental representations to be robust, even in the presence of irrelevant information. Neither PD nor dopamine affected the level of misbinding. This suggests dopamine has very little capacity to affect the corruption of items in WM, a position strengthened by other findings,^26,27^ but see ref. ^28^ However, the generalisability of these findings has not been assessed and other explanations for the findings could not be ruled out.

First, unlike prior investigations of WM in PD patients,^27^ the previous study did not obtain a measure of sensorimotor performance in age-matched controls or patients ON and OFF their medication. Thus, it needs to be demonstrated that patients can have impaired maintenance performance in the absence of any co-occurring sensorimotor deficit. Second, it could also be argued that the results of the previous study^24^ are insufficient to exclude a role for dopamine in short-term recall. The previous assessment of maintenance was sandwiched between tasks that required irrelevant information to be supressed (ignore or update). Thus, some of the apparent deficits of patients on maintenance trials might be explained by switching effects (see ref. ^29^) and/or expectations (which are known to affect the allocation of mnemonic resources).^30^

Third, the maximum maintenance period in the previous study was 6 s. This may be insufficient time for a deficit in the robustness of mental representations to materialise. Though the mechanisms responsible for the effect are debated,^31–33^ it is well known that recall performance can deteriorate with increasing retention periods. Thus, ideally, longer maintenance periods need to be assessed. Finally, to enable the results of the previous study^24^ to be generalised, it is necessary to test WM using an alternative set of parameters. In the previous study, the role of dopamine in affecting spatial WM was not examined. In that investigation the spatial location of the stimuli was irrelevant (did not have to be recalled), but could still influence the results through the obligatory manner in which spatial information consumes mnemonic resources^34,35^ and affects the probability of misbinding events occurring.^36^ This issue may be particularly important in PD, where it has been argued that spatial WM is prominently affected by the disease^37,38^

**Fig. 1:**
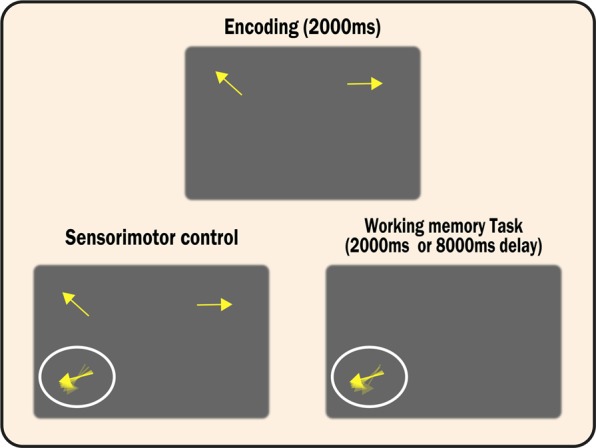
Schematic representation of the task. Participants were presented with two arrows on the left and right side of the screen (2000 ms) and had to retain the orientations of these items. In the memory conditions, a blank screen followed for either 2000 or 8000 ms. After this, a probe item (randomly offset arrow) appeared on the lower left or right side of the screen. Participants had to rotate the probe item until it matched the remembered orientation. The spatial position of the probe indicated which item needed to be recalled, e.g., a probe on the left required the orientation of the left item to be recalled. In the sensorimotor control condition, however, the original stimuli remained on the screen during the probe phase and participants had to simply match the orientations

Here, we seek to examine these hypotheses by testing patients’ pure maintenance abilities in a novel paradigm (Fig. 1). Participants had to remember the orientations of two stimuli presented on the left and right of the screen. After a short (2 s) or long (8 s) delay, one of these items—indicated here by the spatial location of the probe cue—had to be recalled. A ‘zero’ delay condition in which the responses had to be made while the memoranda remained on the screen was also included to rule out sensorimotor confounds.

## Results

Recall was examined in patients using a 2 × 3 repeated measures ANOVA with medication (ON vs OFF) and task (sensorimotor, 2000- and 8000-ms delay) as within-subject factors. This analysis revealed significant effects of task on recall (*F*(2,38) = 76.40, *p* < 0.0001, *ω*^2^ = 0.54), with recall error increasing in both the 8000-ms (*t*(19) = 8.98, *p*_*(Holm)*_ < 0.0001, *d* = 2.00) and 2000-ms (*t*(19) = 9.84, *p*_*(Holm)*_ < 0.0001, *d* = 2.00) delay compared with the sensorimotor condition. Recall error was also significantly impaired for the 8000-ms compared with the 2000-ms delay (*t*(19) = 3.38, *p*_*(Holm)*_ = 0.003, *d* = 0.75). Thus, there was evidence for the decline of memory from the short- to the long-retention periods.

**Fig. 2:**
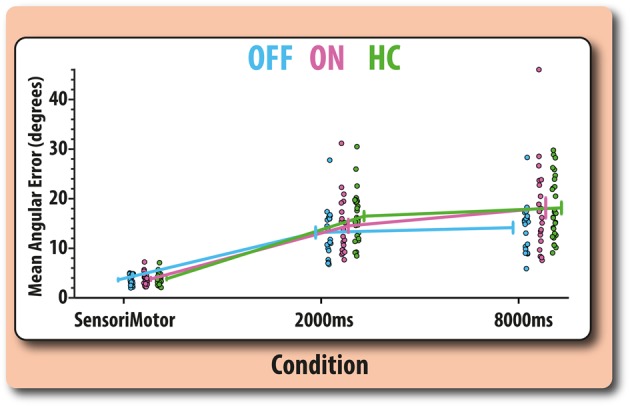
Orientation error recall and sensorimotor control matching performance for patients (split according to medication status: ON vs OFF) and age-matched controls. Error bars (centered on the mean for each condition) reflect the standard error of the mean

Medication state also had a significant main effect on recall (*F*(1,38) = 10.48, *p* = 0.004, *ω*^2^ = 0.039) with patients OFF their dopaminergic medication showing significantly improved recall compared with patients ON their medication. Medication was found to significantly interact with task (*F*(2,38) = 7.17, *p* = 0.002, *ω*^2^ = 0.028). Importantly, there was no significant effect of drug on recall in the sensorimotor condition (*t*(19) = 0.791, *p* = 0.438, *d* = 0.17). To confirm that medication differentially affected performance in the mnemonic conditions, a separate 2 × 2 repeated measures ANOVA was performed with drug (OFF, ON) and delay (2 s, 8 s) as within-subject factors. A significant interaction between drug and delay was found (*F*(1,19) = 6.52, *p* = 0.019, *ω*^2^ = 0.01). This interaction was due to medication significantly impairing recall at the long delay (*t*(19) = 3.49, *p* = 0.002, *d* = 0.78), but having no significant effect on recall for the short delay (*t*(19) = 1.64, *p* = 0.116, *d* = 0.37) (Fig. 2).

There was no significant difference between age-matched controls and patients OFF or ON medication in terms of performance on the sensorimotor task (OFF vs controls, *t*(46) = 0.656, *p* = 0.515, *d* = 0.19; ON vs controls, *t*(46) = 0.086, *p* = 0.93, *d* = 0.025). To contextualise the above medication effects, we compared, separately, the performance of patients OFF and ON their medication with healthy age-matched controls. We performed two separate mixed 2 × 2 ANOVAs with repeated measures on delay (2 or 8 s) and group (the respective patient group or controls) as a between-subject variable. Recall in patients ON their medication was not significantly different compared with age-matched controls (*F* < 1) and group differences in recall did not significantly vary as a function of task (*F*(1,46) = 2.16, *p* = 0.15, *ω*^2^ = 0.003). However, comparison of patients’ performance OFF medication against healthy older adults, revealed significantly better performance in the patient group (*F*(1,46) = 6.26, *p* = 0.016, *ω*^2^ = 0.1). The effect of group did not significantly vary as function of delay (*F* < 1).

In order to deconstruct the cognitive mechanisms behind the effect of medication on recall, we applied a computational model of response selection to the data. First, we examined the effects of medication in PD patients.

### Kappa

This parameter refers to the precision (or concentration of the response orientation around the target orientation) of recall (Supplementary Fig. 1). Medication did not significantly affect kappa values for either short (*W* = 148, *p* = 0.11, *rb* = 0.41) or long delays (*W* = 142, *p* = 0.177, *rb* *=* 0.35).

### Target

Next, we examined the proportion of trials in which the response was centred on the target orientation, i.e. the orientation of the probed item. Medication did not significantly affect the probability for responding to the target orientation for short delay trials (*W* = 129, *p* = 0.388, *rb* = 0.23), but it did significantly increase the probability of responding to the target for long durations (*W* = 166, *p* = 0.021, *rb* = 0.58). Thus, OFF medication patients were more likely to make responses towards the target orientation than when ON medication (Supplementary Fig. 2).

### Misbinding

**Fig. 3:**
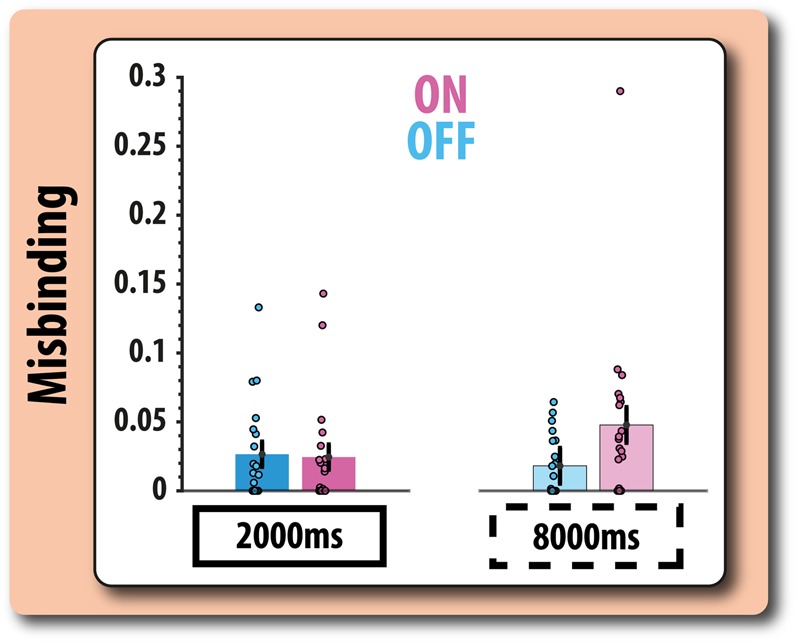
Probability of misbinding ON and OFF dopaminergic medication. Probability of misbinding (reporting the orientation of the non-probed item) according to medication status and delay. Error bars (centred on the mean for each condition) reflect the standard error of the difference between OFF and ON patients for each condition

This parameter captures the extent to which participants are responding to the non-target orientation (the other item participants were remembering but were not asked to recall). This is a measure of the interference of the other item stored in WM on recall performance. There was no significant effect of medication on misbinding for short duration trials (W = 104, *p* = 0.985, *rb* = 0.01), but patients OFF were less likely to make these types of errors than when ON their medication during long-delay trials (*W* = 45, *p* = 0.025, *rb* = 0.57). Thus, medication increased misbinding errors in patients (Fig. 3).

### Guessing

In contrast to misbinding, this parameter reflects the likelihood that participants’ responses are not concentrated on any orientation (Supplementary Fig. 3). Here, unlike the misbinding results, there were no significant effects of medication on guesses, either for short (*W* = 85, *p* = 0.475, *rb* = 0.19) or long durations (*W* = 102, *p* = 0.927, *rb* = 0.029).

In summary, the modelling evidence suggests that patients’ poorer recall when ON their dopaminergic medication, compared with OFF, was due to an increase in the number of misbinding responses. We also undertook some separate analyses to investigate if increased misbinding in patients can also be found using a model-free analysis. Here, we simply looked at the proportion of responses that were closer to the target orientation or non-target orientation. A drug (OFF, ON) by delay (2000 ms, 8000 ms) repeated measures ANOVA revealed that there indeed was a main effect of drug on the proportion of misbinds: patients ON their medication made significantly more responses closer to the non-target than the target orientation (*F*(1,19) = 4.43, *p* = 0.049, *ω*^2^ = 0.021). There was no effect of delay period or interaction between delay period and time (*F*s < 1),

For completeness, we also examined the extent to which these parameters differed between patients (ON and OFF their medication) and controls. Neither patient group (ON or OFF their medication) significantly differed in the level of target nor misbinding response for long-duration trials (see Supplementary Table 3). In line with the overall superior performance of patients OFF medication compared with controls across both delays, there was some evidence that this group of patients showed improvements across multiple parameters of our model compared with controls. Controls, compared with patients OFF, showed a reduction in kappa (lower precision; *p* = 0.038) and higher guessing (*p* = 0.031) rates for the short-duration trials. But these differences were not replicated in the long-delay trials. Thus, cumulatively, when comparing patients OFF with controls, several factors may be responsible for driving the superior performance of patients OFF compared with controls.

### Reaction times

No differences were found for response latency (time to complete responding) between the groups and medication conditions (Supplementary Table 4; Supplementary Materials).

We also examined whether there were any differences in digit span as a function of medication or disease state. There was no significant difference in digit span between patients ON and OFF their medication (*t*(19) = 0.553, *p* = 0.58, *d* = 0.12). Neither group differed in their digit spans compared with controls (OFF vs controls (*t*(46) = 0.70, *p* = 0.491, *d* = 0.20; ON vs controls, *t*(46) = 0.50, *p* = 0.62, *d* = 0.146). Thus, on this clinical measure, there was no evidence that patients (ON or OFF their medication) and controls differed in their basic mnemonic abilities.

## Discussion

This study has revised our understanding of short-term memory abilities in PD. Rather than showing superior performance ON dopaminergic medication, patients’ recall was significantly enhanced, in a delay-dependent manner, when they were OFF medication. Moreover, PD patients OFF their medication were performing significantly better than healthy, age-matched controls. Application of a computational model of response selection^25^ to patients’ pattern of responses revealed that this superior performance by patients OFF dopaminergic medication was due to a decrease in the likelihood of misbinding errors for the longer retention period, that is, they were less likely to report the orientation of the non-probed target orientation. However, the superior performance of patients OFF their medication (compared with controls) appeared to be due to several factors, i.e., improved precision and reduced guessing.

In addition to dopamine, PD is associated with a variety of disturbances to a wide range other neurochemical systems.^12,13,39^ One of the goals of research into patients’ cognitive deficits has been to attempt to fractionate the neural substrates responsible for causing these impairments in order to tailor future interventions.

In order to isolate the causal role that dopaminergic abnormalities have in producing patients’ cognitive deficits, researchers have tested patients ON and OFF their medication. However, previous studies have produced conflicting results, with medication improving neural and behavioural indices of planning, attention and WM,^15,18,40^ but impairing flexible responding to reward and punishment.^7,8^

In attempting to explain these discrepancies, it has been argued that these differential effects of dopaminergic medication are due to the uneven pattern of dopaminergic depletion across fronto-striatal circuits that are separately responsible for cognitive, affective and motoric functions.^9,41,42^ Medication is argued to improve those functions dependent upon the most dopamine- depleted, i.e., dorsal fronto-striatal circuits, but impairs those functions that recruit the less severely affected ventral circuitry. As such, those WM functions that are particularly reliant on dorsal fronto-striatal circuits, such as ignoring and updating,^43,44^ are regularly found to be improved by dopaminergic medication.^45^ However, even within depleted dorsal areas, dopamine levels need to be tightly calibrated to maintain optimal performance. For example, decades of research has revealed that there is an inverted-U shape function linking the level of prefrontal dopaminergic stimulation with WM performance, with either too little or too much dopamine impairing recall.^46–48^

Despite suffering from aberrant signals from the dopamine-depleted dorsal striatum, there is ample evidence to suggest that prefrontal dopamine levels are upregulated in PD patients. Specifically, positron emission tomography (PET) measurements report excess, supranormative, dopamine levels in fronto-parietal areas.^49–51^ Indeed, spatial WM ability—specifically confusing the locations of items across recall episodes—has been argued to display an inverted-U relationship with putative prefrontal dopamine levels in PD patients.^52^ Patients with either putatively low or high dopamine levels were found to make more errors than the group with intermediate dopamine levels.^52^ Thus, it could be hypothesised that the superior performance of PD patients OFF their medication is explained by this group having the task-specific optimal level of dopamine to perform the current task. In contrast, medicated PD patients or healthy age-matched controls, may have diminished performance due to having, respectively, too high or low dopamine levels. The contention that older adults, even in the absence of PD, may have reduced dopamine levels, is supported by research showing that the level of this neurotransmitter declines with age.^53^ Therefore, the performance of controls and medicated PD patients reflects the performance from being situated, respectively, on the left- and right-hand limb of an inverted-U-shape function between dopamine and WM performance.

Improved performance on a WM task in patients OFF their medication has previously been reported.^20^ However, there are some notable differences. Whilst distracter resistance (as indexed with reaction time) in PD patients improved ON medication, digit span was impaired when OFF medication. Based on prior findings that linked distracter resistance to prefrontal activity^54^ and digit span to striatal dopamine,^55^ the authors interpreted this effect as occurring due to an imbalance in the dopamine levels between these regions. However, in the present study we found no evidence for impaired digit span in PD patients as a function of dopaminergic state, perhaps indicating different mechanisms are responsible for generating the effects across these two studies.

Although the suggestion that patients OFF their medication show superior WM abilities due to having optimal dopamine levels to perform the task explains the overall pattern of recall performance, it does not explain why it was misbinding levels that were affected as opposed to precision. Previous studies have not found that PD patients display an increase in misbinding errors and that these types of errors are not modulated by dopaminergic medication.^24,26^ Instead, rather than being affected by disruption to fronto-striatal circuits and the main dopaminergic projections that affect these regions, misbinding is thought to stem from disruption to medial temporal lobe regions. This hypothesis is based upon observations that misbinding levels increase in groups that have, or are at risk of having, compromised hipppcampi (see refs. ^56–60^). One potential reason for the increased sensitivity of misbinding errors to dopaminergic medication in this study is the relatively long retention period that was used in this study. Corruption of recall due to misbinding errors is known to increase with time.^36,61^ Thus, there may have been greater scope for hippocampally mediated processes to disrupt recall in the present experiment.

Importantly, as with the frontal lobe, there is some evidence for heightened activity (as measured with functional magnetic resonance imaging) in medicated PD patients whilst performing cognitive tasks.^62,63^ Thus, it is possible that the absence of medication reduces this excessive activity in the hippocampus. Though it should be noted that this explanation is not mutually exclusive with the above literature on fronto-striatal overdosing in PD patients. There is an extensive and rich set of connections between dopaminergic fronto-striatal and hippocampal regions,^64^ and it may be the disrupted communication between these regions that is responsible for producing the effects observed in the present study. Further research, perhaps combining PET with behavioural techniques such as the one used here, is required to answer this question more definitively.

There may also be a need to revise our conceptualisation of cognitive impairment in PD patients on the basis of these findings. Prominent theories focus on the role of gating information into and out of WM in PD ^65^ usually foveate on this process occurring at encoding or during processing some to-be-updated material. However, the present results suggest that retrieval may also be a dopamine-sensitive gating process, i.e., it could be hypothesised that it is errors at retrieval, rather than maintenance, that are responsible for generating the present results. Future studies will be needed to examine this hypothesis.

Patients OFF their medication also performed consistently better than controls at both delay periods, i.e., their superior performance was not restricted to the long delay conditions. Applying the computational model to the data did not reveal a unique cognitive source of this difference in performance. Differences in kappa (precision) and guessing were apparent at short durations, but these differences were not present to the same extent at longer durations. Thus, it is possible that the overall difference in accuracy (angular error at recall) was driven by multiple factors, which together produce differences in recall. The neurochemical and receptor-specific mechanisms responsible for this effect should be evaluated in future studies by administering relevant agents, e.g., cabergoline to healthy older adults and examining the effect it has on recall.

## Methods

### Study design

This study consisted of 20 patients with PD and 28 age-matched healthy controls (see Table 1 for demographics). Participants were included in this study, if they had normal or corrected-to-normal vision and were not colour blind. Healthy participants were included if they had no history of any neurological or psychiatric illness. All participants gave written informed consent prior to participation. The study was approved and consistent with all relevant local (University of Oxford and National Health Service) regulations (REC no. 14/SC/0044).

**Table 1:** Demographics, questionnaires and baseline cognitive measurements.

Measure	Healthy elderly controls	Parkinson’s disease	Controls versus Parkinson’s disease *P*-value
*n*	28	20	n/a
Age	70.9 (±3.0)	70.5 (±5.8)	0.76
Gender (F/M)	16/12	8/12	0.24^a^
Handedness (R/L)	25/3	16/4	0.37^a^
Hoehn and Yahr stage	n/a	1.95 (±0.2)	n/a
UPDRS motor score ON	n/a	24.0 (±10.6)	n/a
UPDRS motor score OFF	n/a	31.2 (±14.8)	n/a
Change in motor score	n/a	7.3 (±8.5)	n/a
Levodopa equivalent dose (mg/24 h)	n/a	500 (±284)	n/a
Minutes since last dose, ON	n/a	234.1 (±57.3)	n/a
Minutes since last dose, OFF	n/a	962.5 (±233.9)	n/a
Global Cognition (ACE-III score)	95.4 (±4.6)	95.2(±3.4)	0.86
Digit span	21.4 (±3.8)	20.85 (±4.2)	0.63

All values are mean ( ± standard deviation)

*ACE* Addenbrooke’s cognitive examination III, *n/a* not applicable

^a^Chi-squared test

A clinical diagnosis of idiopathic PD (according to the United Kingdom Parkinson’s Disease Society Brain Bank Criteria^66^) was necessary to be eligible to participate in this study. None of the participants were clinically classified as having dementia. Cognitive function was also screened here using the Addenbrooke's Cognitive Examination-III (ACE-III) and digit span (a standard measure of verbal WM). Patients were tested in two morning sessions: ON and OFF their dopaminergic medication (order counterbalanced). Dopaminergic medication levodopa equivalent dose was calculated using a standard algorithm [(Levodopa dose ×1.2 if COMT inhibitor) (×1.2 if 10 mg of Selegiline or ×1.1 if 5 mg of Selegiline)] + [Paramipexole × 400] + [Cabergoline ×160].^67^ See Supplementary Materials for more information (Supplementary Table 2).

#### Working memory task

We used a delayed report task to assess the quality of WM representations. In these designs, participants must reproduce the exact feature of a memoranda, e.g., orientation. This can give an indication of the quality of recall.^68^

In this instantiation, participants were presented with two arrows, either at the top left or right of the screen (2000 ms). Then, in the short-term memory conditions, after either a 2000- or 8000-ms delay, participants were asked to recall the orientation of one of the items. Participants could discern which item had to be recalled according to the spatial location of the probe cue. The probe cue was an arrow presented in the lower left or right of the screen surrounded by a white circle. The spatial location of the cue (left or right) indicated which item had to be recalled, e.g., if the recall cue appeared on the left side of the screen the orientation of the left arrow had to be reproduced.

The initial orientation of the probe cue was randomly offset from the target orientation. Participants had to rotate the arrow clockwise or counter-clockwise (using the ‘A’ or ‘Z’) keys until they were satisfied it matched the target orientation. Participants signified when they finalised their decision regarding their reproduction of the orientation by pressing the ‘Space’ bar. No feedback was presented.

Participants completed 192 trials (64 in each condition). In half of the trials, the probe cue appeared on the left, and on the other half the right. Patients completed the testing on two morning sessions, one ON their usual medication and the other OFF their dopaminergic medication, counterbalanced across drug status. Healthy controls performed one session.

Data were analysed in JASP.^69^ The threshold for statistical significance was set at the conventional level (α = 0.05) and appropriate estimates of effects size are also provided (e.g., Cohen’s d (*d*) for parametric pairwise comparisons, rank biserial correlation (*rb*) for non-parametric contrast and omega squared (*ω*^2^) for ANOVAs). All pairwise comparisons were evaluated using two-tailed tests. Corrections applied to correct the alpha level for multiple comparisons are indicated, e.g., *p*(_*Holm*_),

Quality of recall was assessed using mean angular error (absolute angular difference between orientation of the target and response). Repeated measures ANOVA 2 × 3 was used to examine the effect of the within-subject factor medication (OFF, ON) and condition (sensorimotor control, 2000- and 8000-ms delay). To clarify the results with respect to control performance, two mixed ANOVA 2 × 2 were used to compare patients (ON and OFF) with age-matched controls across delays (2000 and 8000 ms).

Although an accurate measure of the quality of WM, the angular error of recall is composed of distinct components.^68^ We have previously found that the specific type of error participants make on these tasks can be uncovered by applying a mixture model to the data.^26,27^ The specific model^25,70^ decomposes recall into four components, represented in the following equation:

$$p\left( {\hat \theta } \right) = \alpha \phi _\kappa \left( {\hat \theta - \theta } \right) + \mathop {\sum }\limits_{i = 1}^m \beta _i\phi _\kappa \left( {\hat \theta - \varphi _i} \right) + \gamma \frac{1}{{2\pi }},$$

1. Precision (**Kappa**), reflecting the spread of responses around target items (high kappa values reflect better recall; *κ*;).
2. Probability of responding to the target, with higher values indicating a greater likelihood of responding to the target orientation (α).
3. Misbinding parameter, reflecting the probability that the orientation of the non-probed item was reported, with higher values reflect a greater probability of misbinding (β).
4. Guessing parameter indicating the proportion of responses in which there is no systematic relationship between the response orientation and the target orientations (γ).

In the equation, $\hat \theta$ is the responded orientation, $p\left( {\hat \theta } \right)$ is the probability of the responded orientation, $\phi _\kappa$ is a von Mises probability density function centered on zero with concentration *κ*, *m* is the number of incorrect items in the display (in this case 1, indexed by *i*), *θ* is the orientation of the target angle, *φ*_*i*_ is the angle of the incorrect item, and α, β_*i*_, γ are proportions of each component of the response distribution (satisfying $\alpha + {\sum} {\beta _i + \gamma = 1}$). Thus, there are three free parameters, α, β, κ in the model.

As in previous studies,^24^ the models were fit separately for each participant, delay (2000, 8000 ms) and each medication session (for patients). For comparison across medication (OFF, ON), non-parametric analyses (Wilcoxon signed-rank test) were used. For comparisons between patients and controls, Mann–Whitney U tests were employed. Again, as with previous studies, we used the Akaike Information Criterion (AIC) to evaluate whether the model provided a good fit to the data. Across all groups and conditions, the full model (including the misbinding and guessing parameters) provided the best fit of the data (Supplementary Table 1).

### Reporting summary

Further information on research design is available in the Nature Research Reporting Summary linked to this article.

## Supplementary information

Supplementary Material

Reporting Summary

## Data Availability

The experimental data are available from the corresponding author, given reasonable requests.

## Supplementary information

**Supplementary information** accompanies the paper on the *npj Parkinson’s Disease* website (10.1038/s41531-019-0088-2).

## Code availability

The experiment was run and coded in MATLAB 2015b using Psychtoolbox version 3.^71^ The code for running the mixed model is available from https://www.paulbays.com.
