# Supplementary material for: Dopamine affects short-term memory corruption over time in Parkinson’s disease
Source: NPJ Parkinsons Dis. 2019 Aug 5;5:16. doi: 10.1038/s41531-019-0088-2 (PMC6683156; doi:10.1038/s41531-019-0088-2)
Supplement: Supplementary file 1 — Supplementary Material [file 41531_2019_88_MOESM1_ESM.pdf]

Supplementary Materials

Model Evaluation

Model evaluation was done similarly to Fallon et al (2016;17). We compared the model fits (Akaike Information Criterion; AIC) with and without the pertinent model parameters (misbinding and guessing). We did this separately for short (2000ms) and long (8000ms) conditions. In all condition, and for both patients (ON and OFF) and controls, the full model provided the best fit of the data (lowest AIC values).

	Delay	Full model	Full model minus misbinding parameter	Full model minus guessing parameter
**Patients OFF (N= 20)**	2000ms	356	283	651
	8000ms	538	543	908
**Patients ON (N= 20)**	2000ms	562	576	930
	8000ms	1007	1048	1404
**Healthy Controls (N= 28)**	2000ms	1296	1316	1861
	8000ms	1604	1650	2128
**Overall**	2000ms	2214	2275	3443
	8000ms	3149	3241	4440

Supplementary Table 1. Model fits (AIC value) for each model split according to condition and patient group.

Patient Number	l-dopa (mg/day)	Cabergoline (mg/day)	Pergolide (mg/day)	Pramipexole (mg/day)	Ropinirole (mg/day)	Equivalent l-dopa dose
1	300	0	0	0	0	400
2	150	0	0	0	0	704.34
3	150	0	0	0	0	150
4	300	0	0	0	0	300
5	300	0	0	0	0	400
6	1050	0	0	0	4	1130
7	300	0	0	0	5	400
8	250	0	0	0	0	1250
9	600	0	0	0	0	600
10	300	0	0	0	0	400
11	100	0	0	0	0	200
12	450	0	0	0	0	450
13	450	0	0	0	8	610
14	375	0	0	0	0	375
15	300	0	0	0	6	420
16	400	0	0	0	12	640
17	300	0	0	0	12	540
18	187.5	0	0	0	0	287.5
19	400	0	0	0	0	600
20	150	0	0	0	0	150

Supplementary Table 2: Medication regimes for patients.

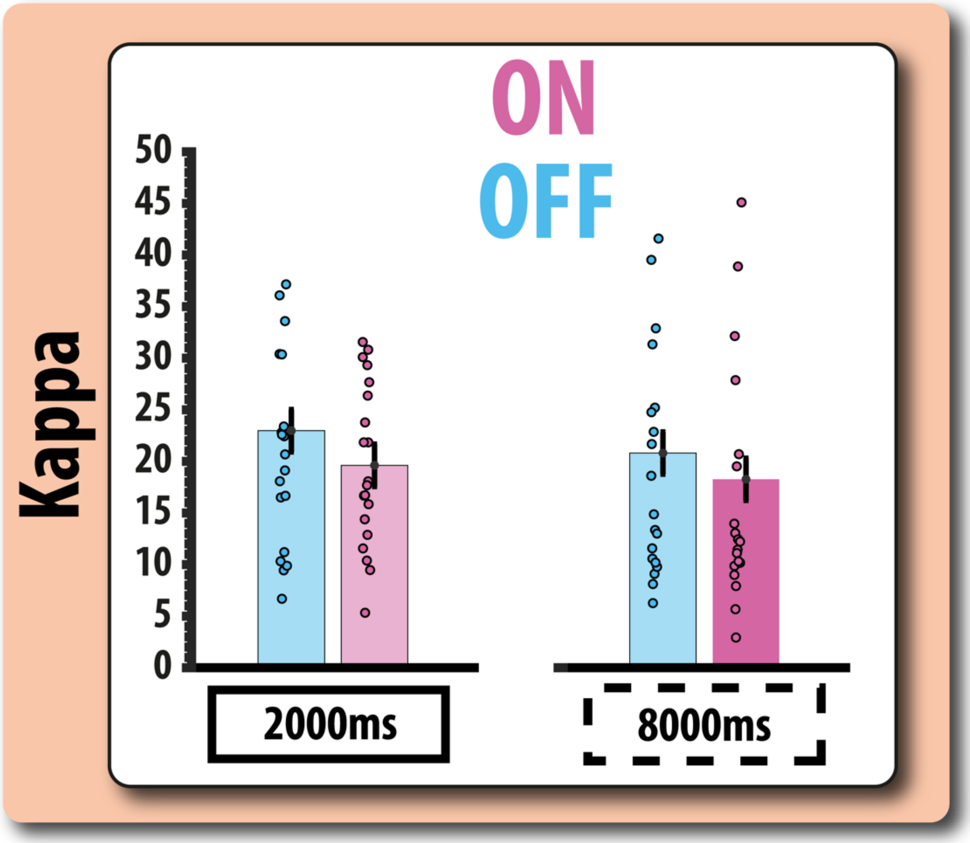

Supplementary Figure 1: Kappa values (precision) for each condition for patients ON and OFF their medication. Error bars (centred on the mean for each condition) reflect the standard error of the difference between OFF and ON patients for each condition.

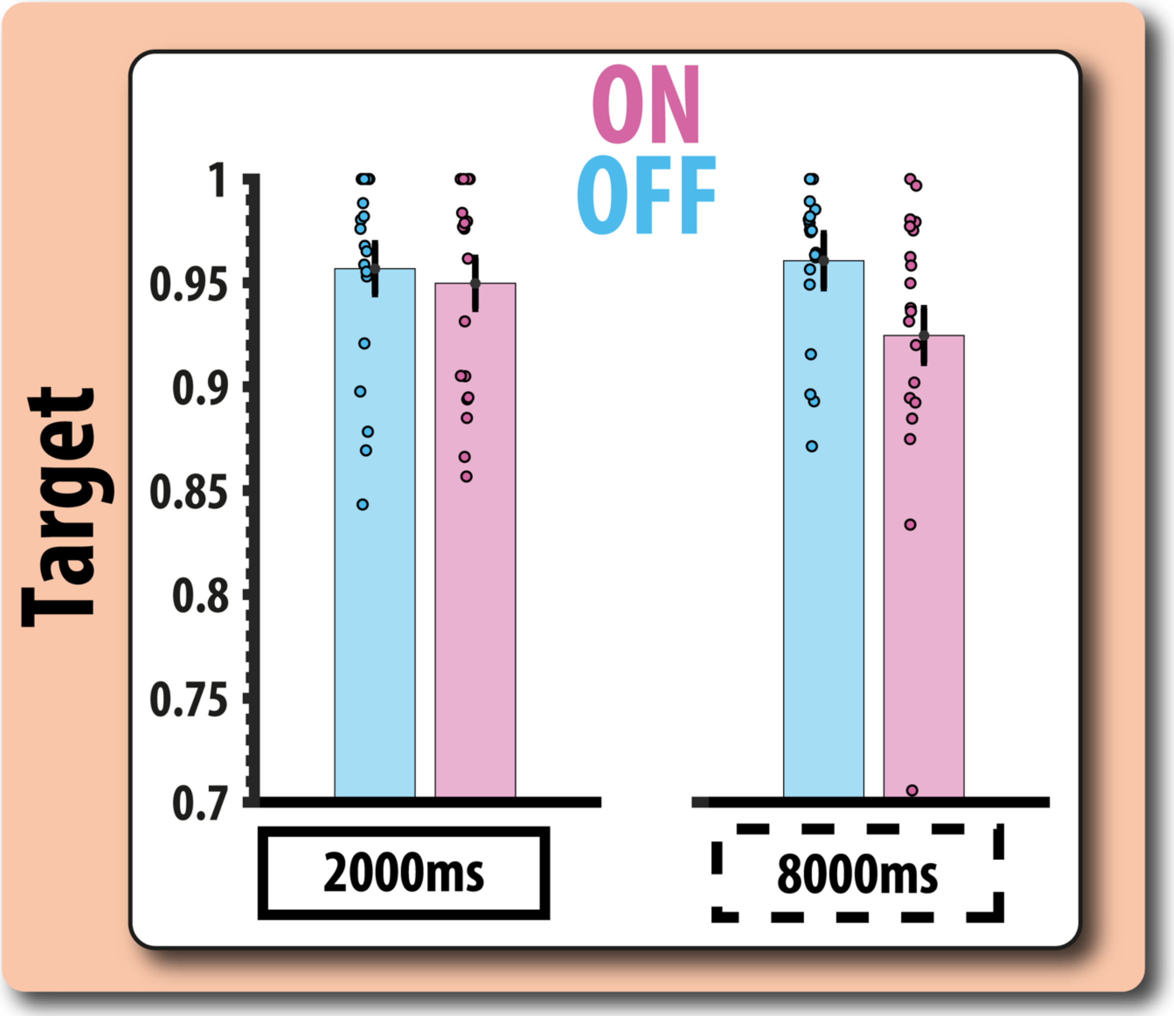

Supplementary Figure 2: Probability of responding to the target orientation in each condition split according to medication status. Error bars (centred on the mean for each condition) reflect the standard error of the difference between OFF and ON patients for each condition.

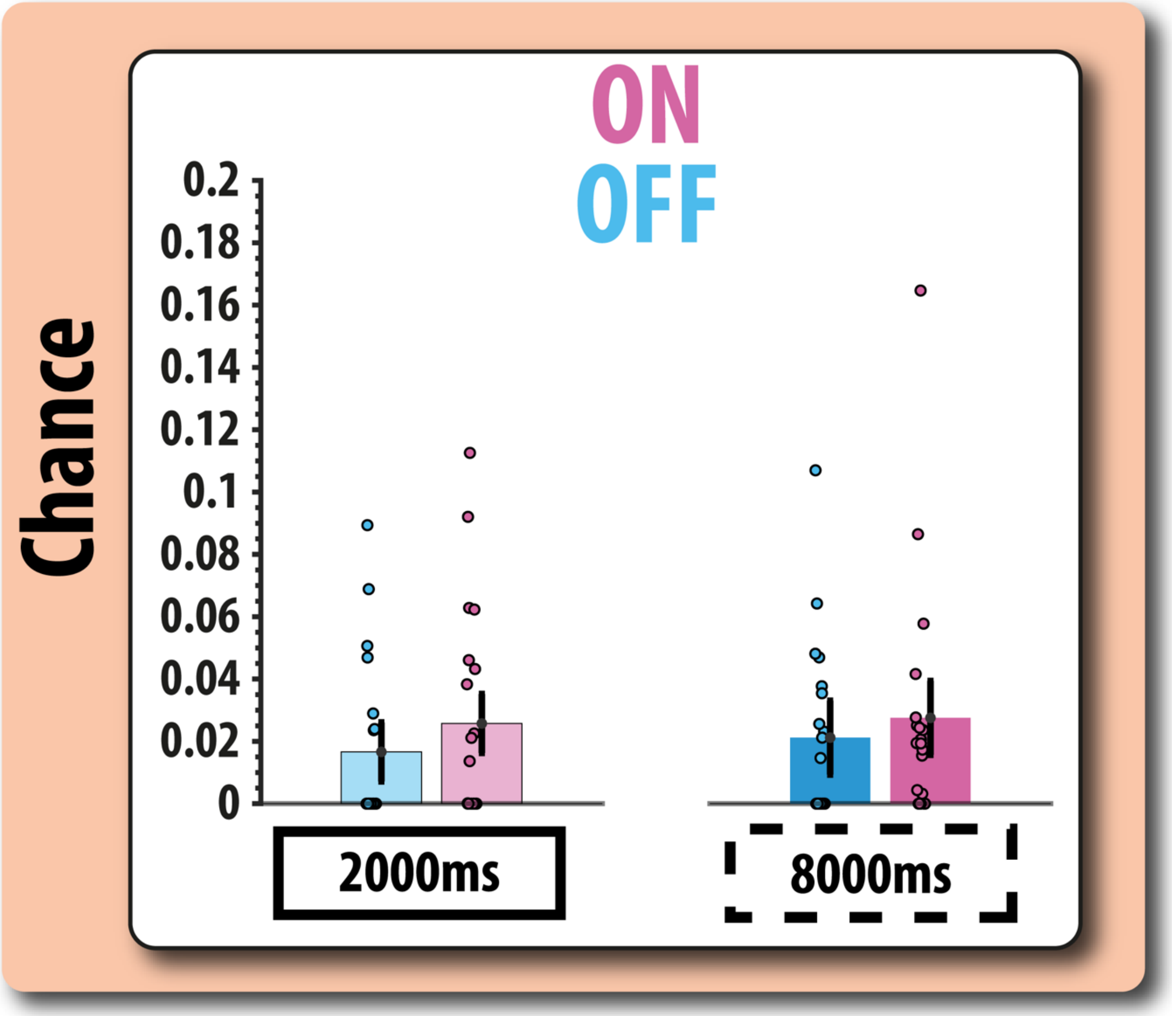

Supplementary Figure 3: Probability of participants making a chance response in each condition split according to medication status. Error bars (centred on the mean for each condition) reflect the standard error of the difference between OFF and ON patients for each condition.

	OFF	ON	HC	OFF vs. HC (p value)	Effect size (OFF-HC)	ON vs. HC (p value)	Effect size (On-HC)
**Kappa**	22.97(12.91)	19.61(7.87)	16.21 (8.73)	.038*	.35	.086	.29
**Target**	.957 (.048)	.95 (.05)	.94 (.046)	.159	.24	.437	.14
**Misbinding**	.027(.036)	.024 (.039)	.025(.029)	.45	.13	.437	.14
**Guess**	.016 (.027)	.025 (.034)	.03 (.03)	.031*	.37	.284	.19

A) Short (2000ms) delay

B) Long (8000ms) delay

	OFF	ON	HC	OFF vs. HC (p value)	Effect size	ON vs. HC (p value)	Effect size (On-HC)
**Kappa**	20.80 (12.71)	18.27 (13.59)	15.36 (7.85)	.202	.218	.884	.03
**Target**	.96(.037)	.924 (.05)	.92 (.07)	.054	.33	.975x	<.01
**Misbinding**	.018 (.02)	.047 (.64)	.035 (.038)	.127	.26	.702	.07
**Guess**	.021 (.028)	.027 (.039)	.04 (.057)	.174	.23	.926	.02

Supplementary Table 3: Table of parameter values (means and SDs) for patients ON and OFF their medication for short (A) and long (B) duration conditions. P values are calculated from Mann-Whitney U. Effect sizes are rank-biserial correlation.

	OFF	ON	HC
**Sensorimotor**	9.01 (2.23)	8.94 (1.96)	8.29(2.20)
**2 second delay**	5.56 (1.66)	5.86 (1.63)	5.94 (1.64)
**8 second delay**	5.94 (1.64)	6.14 (1.45)	5.40 (1.75)

Supplementary Table 4: Means and standard deviations for reaction times (time from the start to end of response period) for each task and group.

Reaction time: Differences in reaction time were not expected. Table S4 shows the descriptive statistics for each condition and group. In order to examine whether there was any evidence for differences in response latency a series of supplementary analyses were performed.

Comparing patients ON and OFF their medication in a repeated measures 2 (ON, OFF) x 3 (control, 2 second delay, 8 second delay) ANOVA did not produce statistically significant differences according to drug (F<1) or significant interaction between drug and condition ($F(2,38) = 1.53$, $p = .229$, $\omega^2 = .001$). There was significant main effect of task ($F(2,38) = 128.86$, $p < .0001$, $\omega^2 = .426$), with participants taking long to respond in the sensorimotor control compared to the 2 and 8 seconds conditions.

Two 2 x 3 ANOVAs were also performed comparing controls to patients (separately for ON and OFF conditions). There was no significant main effect of group for OFF patients compared to controls ($F(1,46) =1.173$, $p = .28$, $\omega^2 = .004$) or patients ON compared to controls ($F(1,46)=1.93$, $p = .17$, $\omega^2 = .019$). There was also no significant interaction between group and task (OFF verses control and ON verses control; *F*s<1).
